# MSCD-YOLO: A Lightweight Dense Pedestrian Detection Model with Finer-Grained Feature Information Interaction

**DOI:** 10.3390/s25020438

**Published:** 2025-01-13

**Authors:** Qiang Liu, Zhongmin Li, Lei Zhang, Jin Deng

**Affiliations:** School of Information Engineering, Nanchang Hangkong University, Nanchang 330063, China; liuqiang20000722@163.com (Q.L.); 2304085401024@stu.nchu.edu.cn (L.Z.); nexuszzz0@outlook.com (J.D.)

**Keywords:** deep learning, dense pedestrian detection, YOLOv8n, Mobile-ViT, SCNeck, DEHead

## Abstract

Pedestrian detection is widely used in real-time surveillance, urban traffic, and other fields. As a crucial direction in pedestrian detection, dense pedestrian detection still faces many unresolved challenges. Existing methods suffer from low detection accuracy, high miss rates, large model parameters, and poor robustness. In this paper, to address these issues, we propose a lightweight dense pedestrian detection model with finer-grained feature information interaction called MSCD-YOLO, which can achieve high accuracy, high performance and robustness with only a small number of parameters. In our model, the light-weight backbone network MobileViT is used to reduce the number of parameters while efficiently extracting both local and global features; the SCNeck neck network is designed to fuse the extracted features without losing information; and the DEHead detection head is utilized for multi-scale feature fusion to detect the targets. To demonstrate the effectiveness of our model, we conducted tests on the highly challenging dense pedestrian detection datasets Crowdhuman and Widerperson. Compared to the baseline model YOLOv8n, MSCD-YOLO achieved a 4.6% and 1.8% improvement in mAP@0.5, and a 5.3% and 2.6% improvement in mAP@0.5:0.95 on the Crowdhuman and Widerperson datasets, respectively. The experimental results show that under the same experimental conditions, MSCD-YOLO significantly outperforms the original model in terms of detection accuracy, efficiency, and model complexity.

## 1. Introduction

Object detection is an important research branch in computer vision, widely applied in various fields such as urban traffic, autonomous driving, and surveillance. However, in its downstream task of dense pedestrian detection, common challenges such as small and densely packed targets with occlusion issues make this task particularly difficult within the broader object detection domain. Traditional object detection algorithms mainly rely on a “handcrafted feature + classification” framework, such as Support Vector Machines (SVM) [1], Histograms of Oriented Gradients (HOG) [2], and Scale-Invariant Feature Transform (SIFT) [3]. However, these methods are heavily feature-dependent, making it difficult to handle multi-scale and pose variations, and they lack end-to-end learning capabilities. In dense crowd scenes, these methods suffer from low accuracy, missed detections, and false detections. Over time, with the continuous development of deep learning, more and more deep learning methods have been applied in object detection. Convolutional neural networks (CNNs) are frequently used in object detection, such as in two-stage algorithms based on region proposals, like the Region-based Convolutional Neural Network (RCNN) series [4,5,6,7], as well as single-stage regression-based algorithms like Single Shot MultiBox Detector (SSD) [8] and the You Only Look Once (YOLO) series [9,10,11,12,13], which have achieved remarkable results. With the emergence of Transformer-based models [14,15,16,17,18], Transformers have been performing increasingly well in object detection tasks, gradually rivaling convolutional neural networks. Whether it is Vision Transformer, Swin Transformer, or their various later variants [19,20,21], they are continuously pushing the benchmarks in object detection tasks.

The two-stage RCNN series has been highly successful in pedestrian detection tasks. Zhang et al. [22] improved detection accuracy by designing a new Aggregation Loss function (AggLoss) and a Partial Occlusion-aware pooling unit (PORoI) based on RCNN. However, this also increased the computational requirements. To achieve faster and more accurate detection, Xu et al. [23] proposed a lightweight model by replacing the backbone network and designing SF-FPN based on Mask-RCNN, improving both model efficiency and accuracy. Zheng et al. [24] enhanced the one-stage SSD algorithm by designing a gated fusion unit (GRU). Experiments showed that the improved GFD-SSD achieved better accuracy compared to Faster-RCNN, but it still could not perfectly balance accuracy and model complexity. Since its introduction, the YOLO model has been widely applied in object detection due to its excellent real-time performance. Zhao et al. [25] improved YOLOv3’s K-means clustering algorithm for better boundary box prediction, achieving improvements in recall, average precision, and F1 score compared to the original algorithm. Wang et al. [26] introduced PB-FPN to improve the neck network of YOLOv5, allowing the network to retain more information during feature fusion and enhancing the representation of small objects. Li et al. [27] improved YOLOv7 by introducing omnidimensional dynamic convolution (ODConv) and a mixed attention mechanism (ACmix), making the model more sensitive to small object detection. Li et al. [28] tackled the problem of false positives and missed detections in UAV detection of small objects by introducing BiFPN, a feature fusion method that models short- and long-range dependencies by adding skip connections between FPN and PAN, reducing information loss and retaining much of the feature information. Lou et al. [29] proposed DC-YOLO, which improved the convolutional architecture of convolutional neural networks by integrating downsampling at different scales, preserving more feature information, and using skip connections to integrate shallow features into deeper layers, improving YOLO’s performance. Wang et al. [30] introduced the BiFormer attention mechanism to more effectively capture relationships between features, and replaced the original loss function with the WIoUv3 loss function, which emphasizes the IoU of small objects, thus improving the model’s performance in small object detection. Currently, many works also focus on enhancing feature representation. Zhang et al. [31] effectively enhanced the feature representation of infrared images by combining frequency domain information feature extraction branches with spatial domain information; additionally, they designed Channel SE-Attention and Position SE-Attention to achieve feature responses between channel information and position information. Zhang et al. [32] designed the Detail Awareness Unit (DAU) and the Contrast Improvement Module (CIM). The DAU integrates multi-scale information, while the CIM is responsible for reconstructing and enhancing details to achieve more powerful feature representation.

Due to the challenges in dense pedestrian detection tasks, such as complex backgrounds, high occlusion rates, small detection targets, and varying scales, these issues significantly affect detection accuracy. To address these problems, current research directions mainly involve designing more complex model architectures, proposing new attention mechanisms or loss functions, and innovatively designing new network modules to adapt to these tasks. Therefore, we propose MSCD-YOLO to tackle the various challenges in pedestrian detection. The main contributions of this paper are as follows:

- We chose to introduce the MobileViT backbone network to replace the backbone network of YOLOv8. By combining local and global feature extraction methods, the extracted features contain more information, while MobileViT’s lightweight structure achieves a reduction in model weight.
- We designed SC-Neck, where the neck network incorporates SPD-Conv for information-preserving downsampling. We also proposed the ReSPD-Conv for upsampling feature maps, achieving an information-preserving neck network through auxiliary upsampling and downsampling. Additionally, CGAFusion is introduced for feature fusion, and the P2 layer is included to improve small object detection performance.
- Finally, we added DEHead to the detection head. By incorporating the EMA attention mechanism for modeling long-distance dependencies and replacing the scale attention mechanism in DyHead while removing the task attention mechanism, we further lightweighted the detection head.

## 2. Materials and Methods

### 2.1. YOLOv8 Algorithm

**Figure 1:**
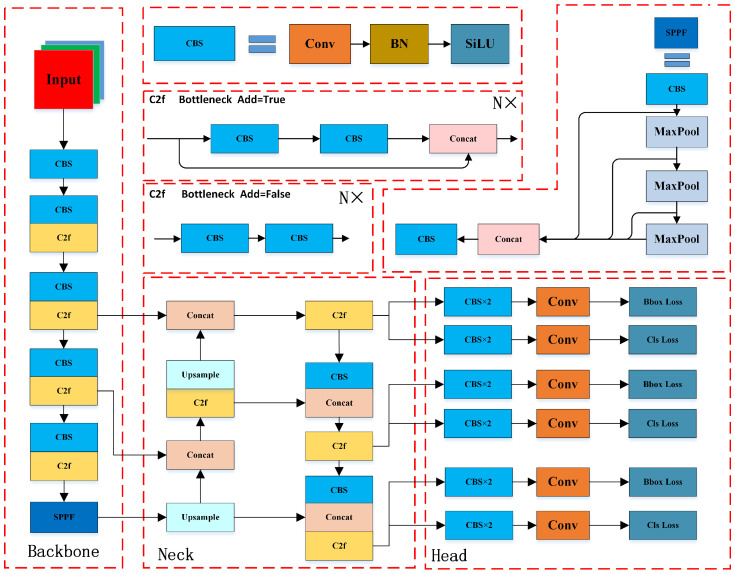
The overall network architecture of YOLOv8.

The YOLOv8 model was proposed by Ultralytics in 2023. Its main architecture is similar to that of YOLOv5, consisting of three parts: the backbone, neck, and head. The structure of the YOLOv8 model is shown in Figure 1. By adjusting the depth and width, five model scales—n, s, m, l, and x—are derived, with each subsequent model being larger. In terms of details, the backbone primarily uses convolution for feature extraction. YOLOv8 replaces YOLOv5’s C3 module and SPP module with C2f and SPPF, respectively, which offer richer gradient flows. The structure of the V8 neck network is fundamentally the same as that of V5, employing an FPN [33] + PAN [34] structure, while replacing the C3 module with the C2f module. Finally, the detection head of YOLOv5 is replaced with a decoupled head, separating the classification and regression tasks, so they no longer share parameters. In this way, it prevents interference between the two tasks, allowing each to achieve better results. Additionally, YOLOv8 adopts a Task Alignment Learning (TAL) dynamic matching strategy instead of the previous IoU matching strategy, enhancing its ability to distinguish between positive and negative samples. Furthermore, it transitions from an anchor-based approach in YOLOv5 to an anchor-free approach, reducing the model’s dependency on prior boxes and improving its learning capability.

### 2.2. MSCD-YOLO Network Model

**Figure 2:**
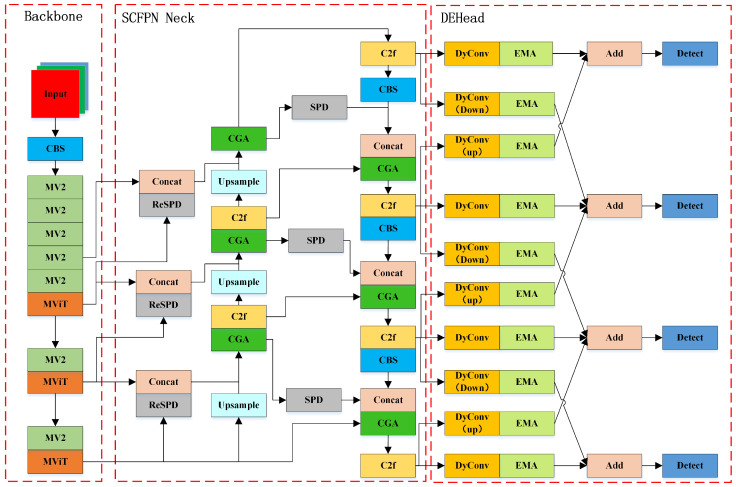
The overall network architecture of MSCD-YOLO.

To better achieve dense pedestrian detection, we focused on common challenges in this task, such as occlusion, small target size, high miss rates, large scale variations, and complex scenes. By conducting an in-depth analysis and research on solutions to these issues, we propose our MSCD-YOLO model, optimizing dense pedestrian detection tasks. The MSCD-YOLO model is based on the YOLOv8n model, and improvements have been made to address various issues specifically for dense pedestrian detection tasks. The structure of the MSCD-YOLO model is shown in Figure 2 below. We replaced the backbone network of YOLOv8 with the lighter MViT, significantly reducing model complexity and allowing for better integration of local and global feature information. The feature fusion network plays a crucial role in the model; the YOLOv8 model uses traditional nearest neighbor sampling or bilinear interpolation for upsampling and strided convolution for downsampling. This method of feature fusion can easily lead to the loss of small target information, and a small receptive field is not suitable for scenarios involving multiple targets, small targets, or occlusion. We designed the SC-Neck to achieve lossless upsampling, downsampling, and richer feature fusion. Additionally, we improved DyHead to DEHead to enhance the model’s detection capability in dense pedestrian detection scenarios, thereby improving the model’s accuracy and stability.

#### 2.2.1. Feature Extraction Network

The backbone network of YOLOv8 is responsible for feature extraction from the input image, consisting of convolution modules and C2f modules. The successive convolution modules perform local feature mapping on the input features, with different convolution kernels representing different receptive field sizes. The C2f module extracts richer features through gradient flow by using multiple consecutive bottlenecks. However, this local feature extraction does not fully capture the information represented by the features. In contrast, Transformer-based models perform global feature mapping on the input features. By applying self-attention operations between each patch and other patches, global features can be effectively captured. However, this approach significantly increases the model’s parameters, greatly reducing the efficiency of the feature extraction network. In 2021, Apple introduced its MobileViT model [35], which effectively combines CNN and Transformer, addressing both the local feature limitations of CNNs and the large parameter size and strict training requirements of Transformers. In this paper, we replaced the backbone network of the YOLOv8 model with MViT to optimize it.

**Figure 3:**
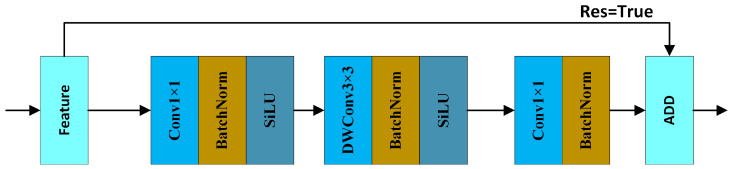
The architecture of MV2.

In MobileViT, the MV2 module is responsible for local mapping with different receptive fields, while the MViT module handles global feature mapping. The MV2 module originates from Apple’s MobileNetV2 [36]. Compared to traditional residual convolution structures, it uses an inverted residual structure. Typically, residual structures shrink feature channels, process features, and then expand them again. However, the lightweight nature of depthwise separable convolution (DWConv), compared to standard convolution, allows the inverted residual structure to avoid increasing the number of parameters. The refinement of channels in the inverted residual structure enables better information propagation through the gradient layers during feature learning. The structure of the MV2 module is shown in Figure 3 below.

The drawback of ViT is that it focuses solely on global features while neglecting the importance of local features, and the significant increase in the number of parameters leads to harsh training conditions. The MViT module addresses these issues by effectively integrating local and global features while using only a small number of parameters.

**Figure 4:**
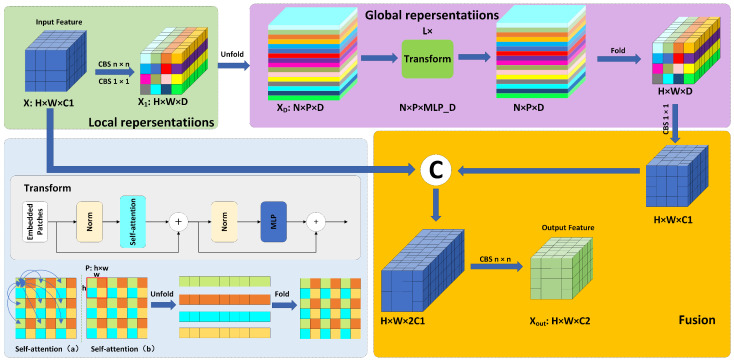
The architecture of MViT Block module.

For the input feature $X\in R^{H\times W\times C}$, where *H* and *W* represent the height and width of the feature, and *C* represents the number of channels, we first use an n×n convolution (usually of size 3×3) for local feature extraction. Then, we use a 1 × 1 convolution to map it to a higher dimension $X_{1}\in R^{H\times W\times D}$. Next, we unfold it to $X_{D}\in R^{N\times P\times D}$ (where *P* is the size of each patch (h×w), and *N* is the number of patches ($\frac{H\times W}{P}$)), and perform lightweight self-attention for global feature extraction. In the example, we only apply transformers to pixels of the same color. It is worth noting that, despite simplifying computations, the initial n×n convolution ensures that each pixel has a receptive field of n×n. Therefore, the effective receptive field for self-attention calculations is still H×W. Compared to the original ViT, this approach reduces redundant computations while maintaining effectiveness, especially in scenarios where adjacent pixel information does not vary significantly. Finally, we fold it back, adjust it with another 1 × 1 convolution, and concatenate it with the original features to prevent gradient vanishing and optimize training. We then perform a final n×n convolution to obtain our output features. The detailed structure of the MViT Block module is shown in Figure 4.

#### 2.2.2. Feature Fusion Network

In the YOLOv8 model, feature fusion still employs FPN combined with PAN while utilizing the C2f module to bring in more gradient flow. This top-down and then reverse operation effectively merges features from different levels and enhances the flow of information from shallow to deep layers. However, in YOLOv8, the upsampling uses nearest neighbor interpolation, and the downsampling employs a 3 × 3 convolution with a stride of 2. It can easily lead to information loss, which is detrimental to subsequent feature fusion.

To address this issue, we focused on SPD-Conv [37], a type of convolution that does not use strides. This non-strided operation helps reduce feature loss during the feature fusion phase. Based on this, we proposed the inverse transformation of SPD-Conv, called ReSPD-Conv, to be used for upsampling. Thus, we achieved non-strided upsampling and downsampling in both FPN and PAN. In the neck network, we designed our SC-Neck using this type of convolution. By adding auxiliary branches alongside the upsampling and downsampling processes in YOLOv8, we ensured that no information is lost. Additionally, we introduced a small target detection layer, P2, and performed CGAFusion for feature integration. This approach allows us to achieve a neck network for feature fusion without losing information. The operational flow of SPD-Conv is shown below.

$$\begin{array}{c} f_{0,0}=X\left[0:S:scale,0:S:scale\right],f_{1,0}=X\left[1:S:scale,0:S:scale\right],\\ \ldots ,f_{scale-1,0}=X\left[scale-1:S:scale,0:S:scale\right];\\ f_{0,1}=X\left[0:S:scale,1:S:scale\right],f_{1,0}=X\left[1:S:scale,1:S:scale\right],\\ \ldots ,f_{scale-1,1}=X\left[scale-1:S:scale,1:S:scale\right];\\ \ldots \\ f_{0,scale-1}=X\left[0:S:scale,scale-1:S:scale\right],f_{1,scale-1}=X\left[1:S:scale,scale-11:S:scale\right],\\ \ldots ,f_{scale-1,1}=X\left[scale-1:S:scale,scale-1:S:scale\right] \end{array}$$

**Figure 5:**
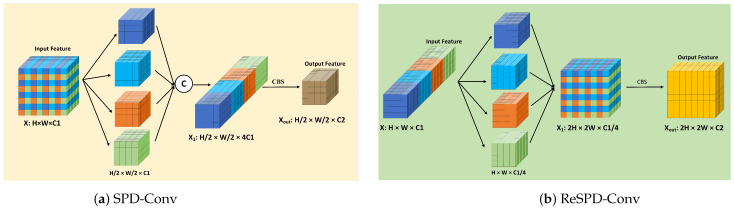
The architecture of SPD-Conv; (**a**) SPD-Conv; (**b**) ReSPD-Conv.

The structure of SPD is shown in Figure 5a, and the structure of ReSPD is shown in Figure 5b. We input a feature $X\in R^{H\times W\times C1}$, slice it, and then concatenate it to form $X_{1}\in R^{H/2\times W/2\times 4C1}$. Next, we use a 1 × 1 convolution to adjust it to the output feature $X_{out}\in R^{H/2\times W/2\times c2}$, eliminating the need for strided convolution operations.

**Figure 6:**
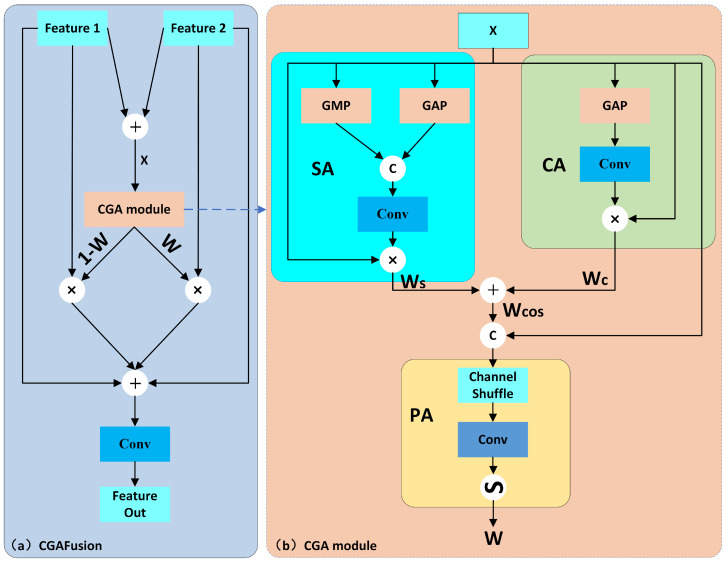
The architecture of CGA feature fusion architecture; (**a**) CGAFusion; (**b**) CGA module.

Since we have constructed an auxiliary feature fusion layer for the original YOLOv8 model, we need to merge the features of the original model with those extracted by the auxiliary layer. We achieve it by introducing the CGAFusion module [38] for feature fusion. The CGAFusion module is illustrated in Figure 6 below. It performs a series of weighted combination operations on two different features $F_{1},F_{2}\in R^{H\times W\times C}$ using the weights obtained from the CGA module to facilitate feature fusion. The formula for CGAFusion is shown in Equation (1), and CGAFusion is illustrated in Figure 6a.

(1) $$F_{fuse}=C_{1\times 1}\left(F_{1}\cdot W+F_{2}\cdot \left(1-W\right)+F_{1}+F_{2}\right)$$

The CGA module uses the input feature $X\in R^{H\times W\times C1}$ to obtain the weights between space and channels. It then applies spatial attention (SA) to obtain $W_{s}$ and channel attention (CA) to obtain $W_{c}$. $W_{cos}$ is obtained by weighting with the input *X*, and then pixel attention (PA) is applied to obtain the final weights *W*. The CGA module is defined by Equations (2)–(4), and the structure of the CGA module is shown in Figure 6b.

(2) $$W_{s}=C_{7\times 7}\left(\left[X_{GAP}^{s},X_{GMP}^{s}\right]\right)$$

(3) $$W_{c}=C_{1\times 1}\left(max\left(0,C_{1\times 1}\left(X_{GAP}^{c}\right)\right)\right)$$

(4) $$W=\sigma (C_{7\times 7}\left(CS\left(\left[X_{1},W_{\cos}\right]\right)\right))$$

#### 2.2.3. Detection Head

The detection head in YOLOv8 has been updated to a decoupled head compared to previous generations of YOLO. It separates the classification task from the regression task, meaning the two no longer share parameters, allowing them to operate independently and better fulfill their respective tasks. However, the challenges faced in dense pedestrian detection, such as small objects, occlusion, and varying object scales, prompted us to introduce DyHead and make targeted improvements to design our DEHead.

**Figure 7:**
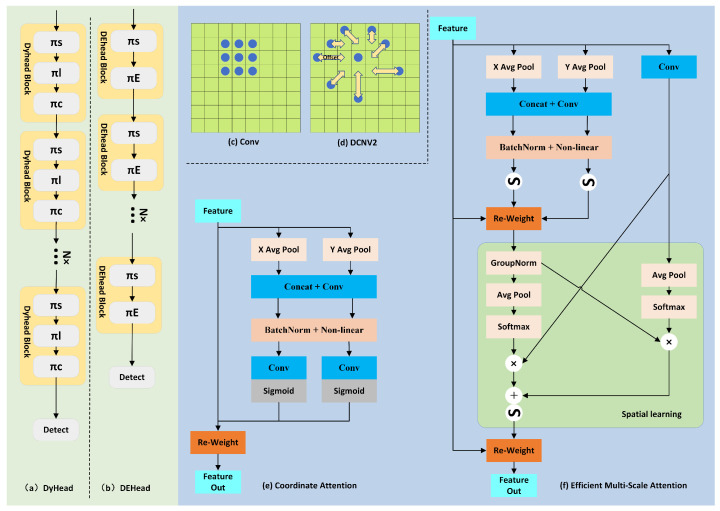
The improvement of Head; (**a**) DyHead; (**b**) DEHead; (**c**) Conv; (**d**) Deformable Conv; (**e**) coordinate attention; (**f**) Efficient Multi-Scale attention.

DyHead [39], introduced by Microsoft in 2021, is composed of stacked DyHead blocks. Each DyHead block includes spatial attention, scale attention, and task attention mechanisms. These mechanisms perform scale-aware, spatial-aware, and task-aware operations at different levels, while integrating features across different levels to improve sensitivity to features related to small objects. The spatial awareness operation $\pi_{S}$ is carried out using DCNV2, which adjusts the receptive field to achieve spatial perception. Figure 7c,d show how DCNV2 enlarges the receptive field compared to standard convolution. In dense pedestrian detection, there is only one class, so we removed the task-aware operation $\pi_{L}$ from DyHead. Additionally, we replaced the scale attention mechanism $\pi_{C}$ with the EMA attention mechanism (Efficient Multi-Scale attention) $\pi_{E}$ to enhance the detection head’s ability to detect small objects. The formulas for DyHead and DEHead are provided, and the structures of DyHead Block and DEHead Block are shown in Figure 7a,b.

(5) $$W\left(F\right)=\pi_{C}\left(\pi_{S}\left(\pi_{L}\left(F\right)\cdot F\right)\cdot F\right)\cdot F$$

(6) $$W\left(F\right)=\pi_{E}\left(\pi_{S}\left(F\right)\cdot F\right)\cdot F$$

The EMA [40] builds upon its predecessor, the Coordinate Attention (CoorA) [41]. Generally, we believe that global average pooling can be used to extract channel information, as seen in the SE attention mechanism. The CoorA further integrates spatial information across dimensions into the extracted channel information, making it particularly suitable for enhancing feature fusion. The CoorA performs one-dimensional global pooling on the input features $X\in R^{H\times W\times C}$ in both the height (H) and width (W) directions to obtain $X_{1}\in R^{1\times W\times C}$ and $X_{2}\in R^{H\times 1\times C}$. Then, the obtained $X_{1}$ and $X_{2}$ undergo concatenation and convolution operations for information extraction. Afterward, batch normalization (BatchNorm), activation functions (Sigmoid), and other processing steps are applied. This process effectively preserves information that can focus on pixel-level targets. Building upon this, EMA introduces an additional branch that interacts with the features modeled by the CoorA, allowing for effective information exchange between shallow and deep layers. This global modeling can provide excellent pixel-level attention for images. The structures of the CoorA and the EMA are illustrated in Figure 7e,f.

## 3. Results

### 3.1. Experimental Environments and Dataset

The configuration of the experimental environment is as follows. The operating system is Ubuntu 20.04, the GPU is a 24GB RTX 3090, the CPU is an Intel Xeon Platinum 8362, the Python version is 3.8, and the PyTorch framework is torch 1.13.1 + cu117. The training image size is uniformly set to 640 × 640, the number of training epochs is 100, the optimizer chosen is SGD, and the training batch size is set to the maximum batch size with default hyperparameters. The experimental environment configuration is shown in Table 1 below.

To verify the generalization ability and accuracy of the model, the datasets we selected must contain dense detection targets, significant occlusion, and variable scenes. In this way, we can effectively validate the proposed model.

There are many existing pedestrian detection datasets, such as Caltech-USA, KITTI, Cityperson [42], and COCOPerson, but these datasets suffer from issues like having few targets, single scenes, and limited scales. We validate our model using two different public datasets, Crowdhuman [43] and Widerperson [44]. Comparing across different datasets, it can effectively verify the model’s generalization ability and accuracy.

First, the Crowdhuman dataset contains 15,000 training images, 5000 test images, and 4370 validation images. The training and validation sets include at least 470k instances, with approximately 23 targets per photo. It encompasses a wide variety of scenes, such as roads, parks, indoor environments, and construction sites, with a predominance of small and occluded targets, making it very suitable for our experimental validation.

Next, Widerperson is another commonly used dataset for dense pedestrian detection, containing 8000 training images and 1000 validation images, with a total of 240k targets. The average number of targets per image reaches 29.51, making it the dataset with the highest density among currently available public datasets. It also has rich scene variations and target scale diversity, making it very suitable for this experimental validation. Table 2 provides a comparison of the different datasets.

### 3.2. Evaluation Indexes

The evaluation metrics selected in this paper are model parameters (Param), mean average precision (mAP), and recall (R). The model parameters (Param) serve as an indicator of whether a model is lightweight, and the recall (R) effectively reflects the rate of missed detections in the model. The calculation of recall is shown in Equation (8).

(7) $$P=\frac{TP}{FP+TP}$$

(8) $$R=\frac{TP}{FN+TP}$$

In the equation, the parameter TP (True Positives) represents the number of correct detections, FP (False Positives) represents the number of incorrect detections, and FN (False Negatives) represents the number of missed detections. Three parameters form the basis for the calculation of recall.

Mean average precision (mAP) is a metric that effectively reflects the accuracy of the model. It is composed of precision (P) and recall (R), and its calculation is shown in Equation (10). By setting different IoU thresholds (such as IoU at 0.5 or IoU from 0.5 to 0.95), we can obtain the values of mAP@0.5 and mAP@0.5:0.95. The specific calculation method is as follows.

(9) $$AP_{i}=\int_{0}^{1}P_{i}\left(R_{i}\right)dR_{i}=\sum_{k=0}^{n}P_{i}\left(k\right)\Delta R_{i}\left(k\right)$$

(10) $$mAP=\frac{1}{m}\sum_{i=1}^{m}AP_{i}$$

### 3.3. Experimental Results and Analysis

#### 3.3.1. Ablation Experiments

**Figure 8:**
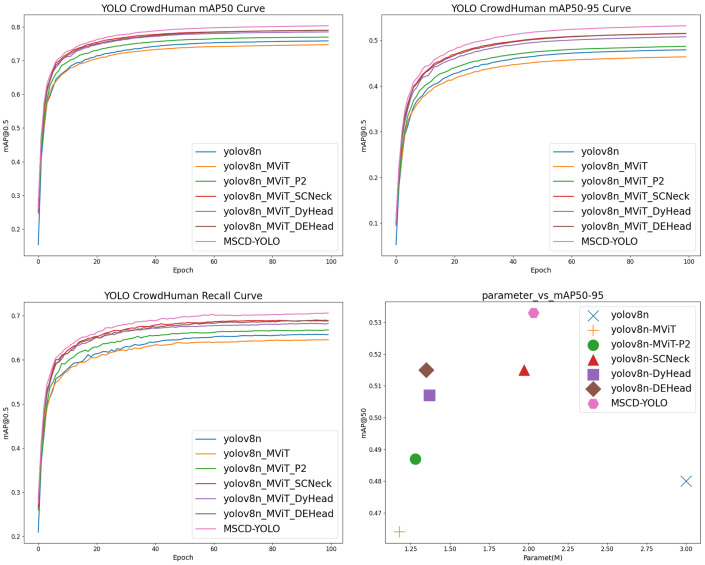
Results of the ablation experiment on the Crowdhuman datasets.

**Figure 9:**
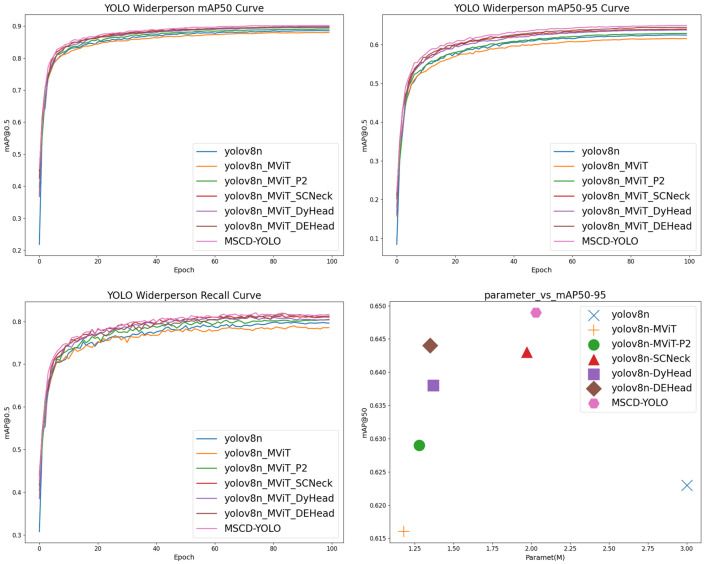
Results of the ablation experiment on the Widerperson datasets.

We conducted ablation experiments to analyze the effectiveness of our improved modules by adding different improvement modules to the YOLOv8n model (Baseline) and evaluating their performance. We refer to the baseline as YOLOv8n. The experimental result of replacing the backbone network is denoted as YOLOv8n_MViT. The result of adding the small target layer is denoted as YOLOv8n_MViT_P2. Replacing the neck network with our SCNeck is denoted as YOLOv8n_MViT_SCNeck, and replacing the detection head with DyHead and our DEHead is denoted as YOLOv8n_MViT_DyHead and YOLOv8n_MViT_DEHead. Finally, combining all the improved modules results in our final model, named MSCD-YOLO. All models were trained with the default parameters for 100 epochs. The experimental results on the Crowdhuman dataset are shown in Table 3 and Figure 8, and the results on the Widerperson dataset are shown in Table 4 and Figure 9.

From the results, we can see that although replacing the backbone network leads to a slight loss in accuracy, the model with the replaced backbone has only one-third of the parameters compared to the baseline YOLOv8n. Due to MViT’s efficient local and global feature extraction capabilities, combined with the addition of a small object layer, the model surpasses the baseline with minimal additional cost. This gives us a lot of flexibility for subsequent improvements. After replacing the backbone with MViT and adding SCNeck, the finer-grained feature interaction in the neck network effectively integrates low-level positional information with high-level semantic information. This enables the model to better judge edge information. We achieved various accuracy gains. On the Crowdhuman dataset, recall (R) and mAP50, mAP50-95 increased by 2.9%, 3.5%, and 3.5%, respectively; on the Widerperson dataset, recall (R), mAP50, and mAP50-95 increased by 1.6%, 1.5%, and 2.0%, respectively, while bringing only a small increase in parameters. After replacing SCNeck with DyHead, DyHead’s multi-scale interaction mechanism better captured multi-scale information, enabling the model to detect and recognize objects of different sizes and shapes. On the Crowdhuman dataset, recall (R), mAP50, and mAP50-95 increased by 2.3%, 2.7%, and 2.7%, respectively; on the Widerperson dataset, recall (R), mAP50, and mAP50-95 increased by 0.7%, 1.2%, and 1.5%. Next, after replacing DyHead with DEHead, replacing DyHead with DEHead retained DyHead’s ability to interact with multi-scale information while introducing long-range semantic modeling, resulting in deeper semantic information and a more focused perception capability for the model. On the Crowdhuman dataset, recall (R), mAP50, and mAP50-95 increased by 3.2%, 3.4%, and 3.5%, respectively; on the Widerperson dataset, recall (R), mAP50, and mAP50-95 increased by 1.6%, 1.5%, and 2.1%, showing improvements over DyHead. In comparison to the baseline YOLOv8n, the model’s complexity is less than half. Finally, combining all the improvements into MSCD-YOLO, while the model complexity is only two-thirds that of YOLOv8n, recall (R) on the Crowdhuman dataset improved by 4.5%, mAP50 by 4.6%, and mAP50-95 by 5.3%; on the Widerperson dataset, recall (R) improved by 1.9%, mAP50 by 1.8%, and mAP50-95 by 2.6%.

#### 3.3.2. Comparison and Analysis of Different Model

**Figure 10:**
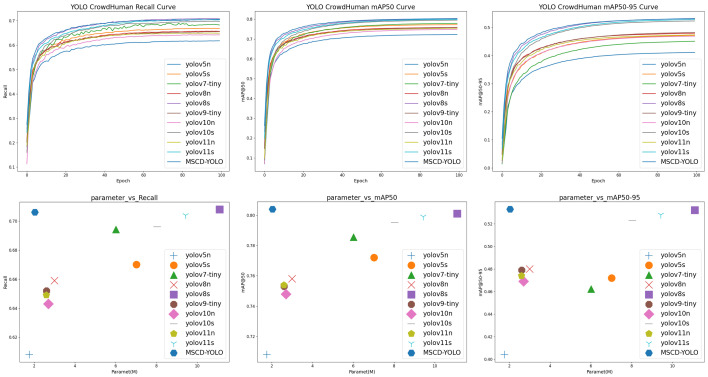
Comparison of different models of mAP@0.5, mAP@0.5-0.95, Recall, Param on the Crowdhuman datasets.

**Figure 11:**
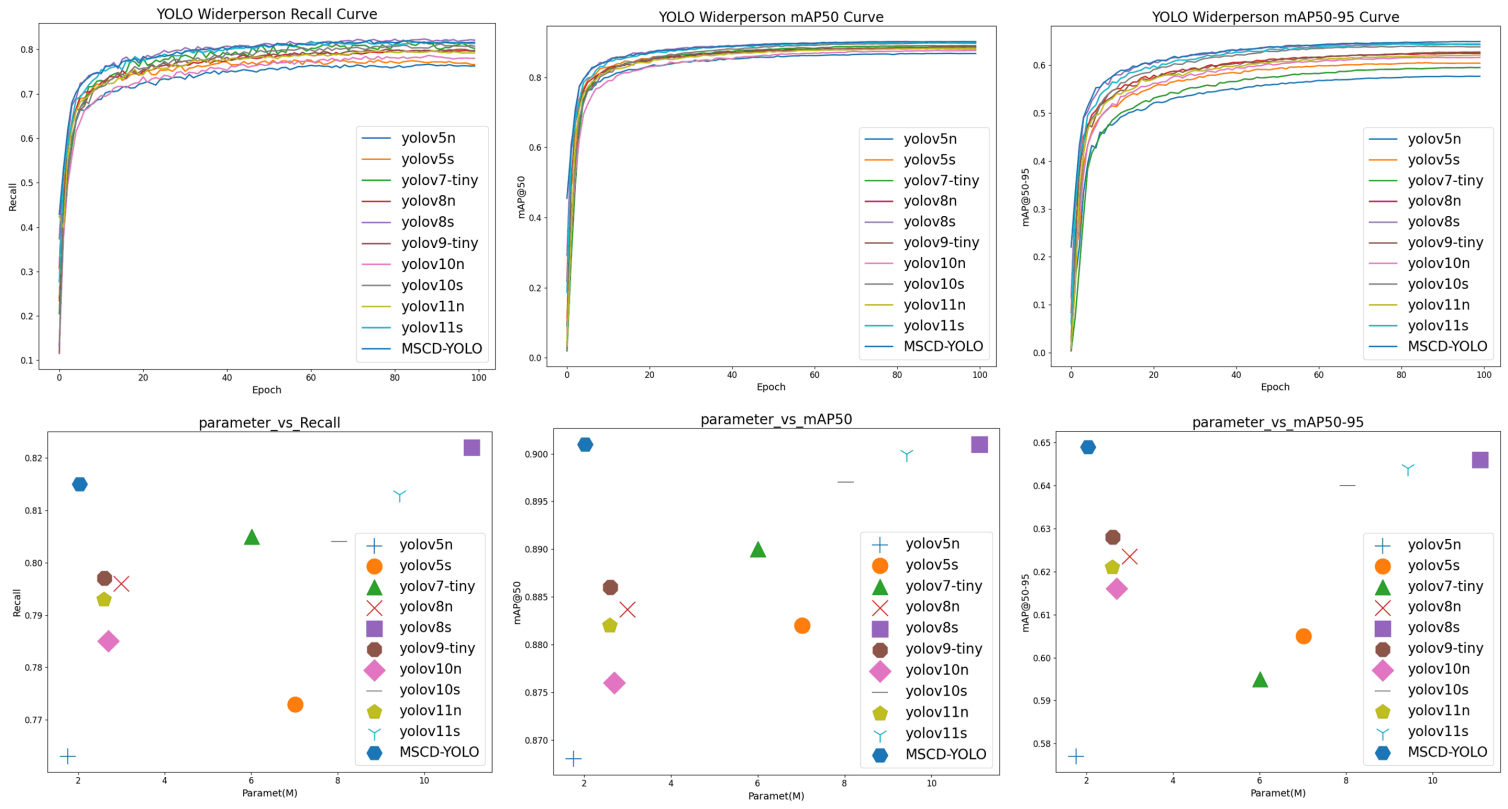
Comparison of different models of mAP@0.5, mAP@0.5-0.95, Recall, Param on the Widerperson datasets.

We conducted an effective analysis of the models by comparing one-stage deep learning models like SSD and the YOLO series with two-stage deep learning models such as the RCNN series. All models were trained for 100 epochs with consistent training parameters and without using pretrained weights. The final results are shown in Figure 10 and Figure 11, Table 5 and Table 6.

From the experimental results, we can see that our model achieves an excellent balance between complexity and accuracy. The results show that the model with the smallest complexity is YOLOv5n, with only 1.76M parameters. In comparison, our model adds only 0.27M parameters, indicating that in terms of model complexity, our model is firmly in the top tier alongside YOLOv5n. Regarding the recall rate, our model ranks just behind YOLOv8s, but with only 1/5 of YOLOv8s’s parameter count, the difference is merely 0.4%, demonstrating the model’s outstanding performance. In terms of mAP@0.5 and mAP@0.5:0.95 accuracy, our model holds a clear lead. Whether compared to a complex two-stage model or the one-stage model, our model achieves the best results with minimal parameters.

#### 3.3.3. Visual Analysis of Results

**Figure 12:**
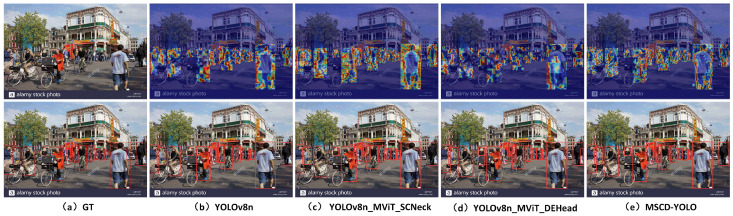
Comparison of feature extraction between different models (street).

**Figure 13:**
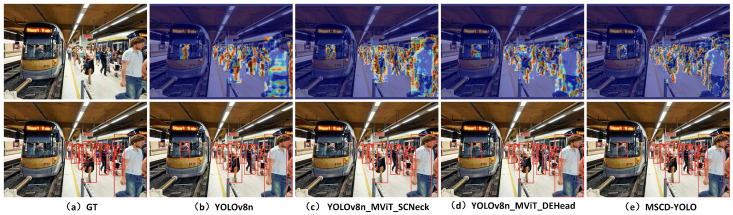
Comparison of feature extraction between different models (train).

**Figure 14:**
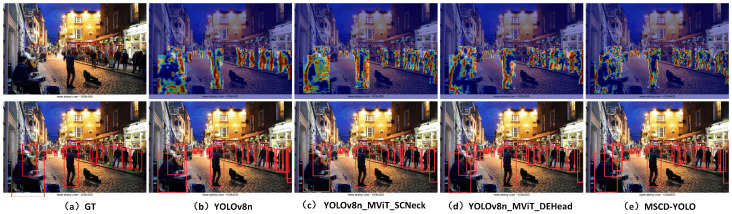
Comparison of feature extraction between different models (night).

**Figure 15:**
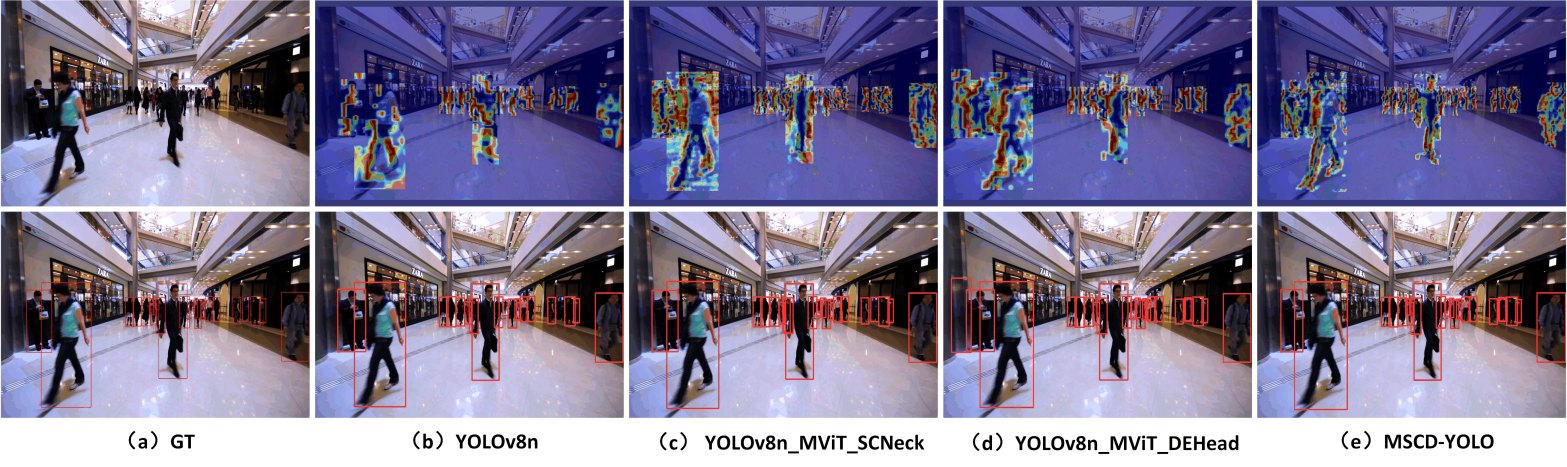
Comparison of feature extraction between different models (mall).

We conducted feature heatmap visualization to compare the MSCD-YOLO model with the baseline YOLOv8n model to better demonstrate the performance of our model. Feature heatmap visualizations and model inference were performed in various scenes, such as crowded streets during the day, train stations, poorly lit roads at night, and indoor shopping malls. These diverse scenarios, shown in Figure 12, Figure 13, Figure 14 and Figure 15, effectively validate the generalization capability and outstanding performance of our model. Figure 12a represents the image with original labels, Figure 12b shows the feature extraction heatmap and model inference results of the YOLOv8n (Baseline) model, and Figure 12c–e present the feature extraction heatmap and model inference results of the different improvements to the baseline model. Figure 13, Figure 14 and Figure 15 follow the same structure.

From the feature heatmap visualization results (heatmap distribution) and model inference, it can be observed that, compared to the heatmaps of the YOLOv8n model, the integration of SCNeck enables higher-quality information fusion, helping the model focus more on edges. Additionally, the long-range modeling capability of DEHead enriches the semantic information of features, allowing the model to concentrate its attention more effectively. The combination of these two components in the MSCD-YOLO model enables it to focus on the entire person, including actions, movements, and full-body detection, resembling the way the human brain perceives pedestrians. This characteristic highlights the holistic correlation of pedestrians. Compared to the YOLOv8n model, the MSCD-YOLO model demonstrates more precise attention to pedestrians, enhancing its detection capabilities and effectively distinguishing between pedestrian and non-pedestrian regions. Our heatmaps more accurately cover pedestrians without being influenced by other objects. For example, in Figure 12b, due to crowd occlusion, the YOLOv8n model misses detections on the right side of the image. In Figure 13a, although the labels do not include parts of pedestrians obscured by glass, YOLOv8n mistakenly detects two targets and has multiple missed detections on the right side of the image. In contrast, MSCD-YOLO correctly detects these targets and accurately identifies different targets in dense crowd areas. Figure 14 and Figure 15 represent low-light nighttime scenes and indoor environments, respectively. In these scenarios, the YOLOv8n model performs poorly. For instance, in Figure 14b, there are many false detections in the darker areas on the right, while in Figure 15b, there are several missed detections in the right and central regions. The miss rate of YOLOv8n is significantly higher in low-light environments than in well-lit daytime scenes. In contrast, the MSCD-YOLO model performs much better. It can even detect targets not included in the labels, such as the person on the electric scooter on the left and the distant small pedestrian targets in Figure 14e, as well as partially occluded targets detected in the shadowed area on the right.

## 4. Discussion

Compared to the original YOLOv8n model, the proposed MSCD-YOLO model has improved detection accuracy for dense person detection. However, there are still some minor issues, such as the difficulty in detecting very concealed targets and the cases of missed detections in situations with significant occlusion. For instance, when only a part of the body is exposed, the detection performance is improved, but missed detections still occur. These challenges pose significant obstacles to our model’s detection capabilities. In the future, we hope to continue optimizing the model, such as by incorporating new attention mechanisms. Additionally, although our model is smaller, the specially designed SCNeck leads to a more multi-scale and unique feature fusion method, which inevitably increases inference time. We can reduce inference time while ensuring detection performance by pruning the model, distilling it, reparameterizing, or adopting more innovative Neck fusion layers. At the same time, since the annotations in the Widerperson dataset are not as strong as those in the Crowdhuman dataset, and Widerperson has greater scene variations with a smaller data scale, the model’s improvement on the Widerperson dataset is not as noticeable as on Crowdhuman. To achieve higher accuracy, we can add extra supervision heads or design new attention mechanisms that help the model focus more on key areas.

## 5. Conclusions

This paper introduces a model called MSCD-YOLO, specifically designed for dense pedestrian detection tasks. It is capable of handling scenarios with dense crowds, occlusion, varying target scales, complex scenes, and a large number of targets. First, to address model complexity, we incorporate MobileViT, which combines local features extracted by CNN with global features obtained by Transformers, thereby increasing the amount of information extracted by the backbone network. Then, to tackle the common challenges in dense pedestrian detection tasks, we propose our SCNeck. This introduces stride-free convolution (SPD) and its reverse operation, ReSPD, creating new auxiliary branches and fusing them with the original neck using CGAFusion. This approach solves the issue of information loss during feature fusion. In addition, we improve the DyHead module to better adapt it for dense pedestrian detection tasks, enabling it to detect small targets more effectively. To evaluate our model, we conducted experiments on two different datasets, Crowdhuman and Widerperson. This approach allows us to not only test the model’s performance but also compare its generalization capabilities. The experimental results demonstrate that our model holds a clear advantage over the current mainstream models.

## Figures and Tables

**Table 1:** Experimental platform.

Environment	Configuration
Operating System	Ubuntu 20.04
GPU	3090 (24 GB)
CPU	Intel Xeon Platinum 8362
Python	3.8.19
Deep Learning Framework	torch 1.13.1 + cu117
Optimizer	SGD

**Table 2:** Dense pedestrian detection dataset.

Datasets	Number of Images	Target Instances	Target Instance
Caltech-USA	42,782	13,674	0.32
KITTI	3712	2322	0.63
COCOPerson	64,115	257,252	4.01
Cityperson	2975	19,238	6.47
Crowdhuman	15,000	470,000+	22.63
Widerperson	8000	240,000	26.51

**Table 3:** Ablation experiment on the Crowdhuman datasets.

Baseline	MViT	MViT (P2)	SCNeck	DyHead	DEHead	Param (M)	R (%)	mAP50 (%)	mAP50-95 (%)
YOLOv8n						3.0	65.9	75.8	48.0
YOLOv8n_MViT	√					1.18	64.5	74.7	46.4
YOLOv8n_MViT_P2		√				1.28	66.7	77.1	48.7
YOLOv8n_MViT_SCNeck		√	√			1.97	68.8	79.3	51.5
YOLOv8n_MViT_DyHead		√		√		1.37	68.2	78.5	50.7
YOLOv8n_MViT_DEHead		√			√	1.35	69.1	79.2	51.5
MSCD-YOLO		√	√		√	2.03(−0.97)	70.4(+4.5)	80.4(+4.6)	53.3(+5.3)

**Table 4:** Ablation experiment on the Widerperson datasets.

Baseline	MViT	MViT (P2)	SCNeck	DyHead	DEHead	Param (M)	R (%)	mAP50 (%)	mAP50-95 (%)
YOLOv8n						3.0	79.6	88.3	62.3
YOLOv8n_MViT	√					1.18	78.4	88.0	61.6
YOLOv8n_MViT_P2		√				1.28	80.4	89.1	62.9
YOLOv8n_MViT_SCNeck		√	√			1.97	81.2	89.8	64.3
YOLOv8n_MViT_DyHead		√		√		1.37	80.3	89.5	63.8
YOLOv8n_MViT_DEHead		√			√	1.35	81.2	89.8	64.4
MSCD-YOLO		√	√		√	2.03(−0.97)	81.5(+1.9)	90.1(+1.8)	64.9(+2.6)

**Table 5:** Comparison of different models on the Crowdhuman datasets.

Method	Param (M)	R (%)	mAP50 (%)	mAP50-95 (%)
Faster-RCNN	41.34		78.0	49.9
Mask-RCNN	43.99		77.0	47.0
SSD	23.746		69.6	34.7
YOLOv5n	1.76	60.8	70.8	40.4
YOLOv5s	7.02	67.0	77.2	47.2
YOLOv7-Tiny	6.01	69.41	78.56	46.22
YOLOv8n(Baseline)	3.0	65.9	75.8	48
YOLOv8s	11.1	70.8	80.1	53.2
YOLOv9-Tiny	2.61	65.2	75.3	47.9
YOLOv10n	2.7	64.3	74.8	46.9
YOLOv10s	8.03	69.6	79.5	52.3
YOLOv11n	2.59	64.9	75.4	47.4
YOLOv11s	9.41	70.4	79.8	52.8
**MSCD-YOLO(Ours)**	2.03	70.6	80.4	53.3

**Table 6:** Comparison of different models on the Widerperson datasets.

Method	Param (M)	R (%)	mAP50 (%)	mAP50-95 (%)
Faster-RCNN	41.34		86.9	58.9
Mask-RCNN	43.99		86.9	59.0
SSD	23.746		77.9	43.7
YOLOv5n	1.76	76.3	86.8	57.7
YOLOv5s	7.02	77.3	88.2	60.5
YOLOv7-Tiny	6.01	80.5	89.0	59.5
YOLOv8n(Baseline)	3.0	79.6	88.3	62.3
YOLOv8s	11.1	82.2	90.1	64.6
YOLOv9-Tiny	2.61	81.9	88.6	62.8
YOLOv10n	2.7	78.5	87.6	61.6
YOLOv10s	8.03	80.4	89.7	64.0
YOLOv11n	2.59	79.3	88.2	62.1
YOLOv11s	9.41	81.3	89.9	64.4
**MSCD-YOLO(Ours)**	2.03	81.5	90.1	64.9

## Data Availability

You can access the Crowdhuman and Widerperson datasets through the following links. Crowd Human dataset: https://www.crowdhuman.org/ (accessed on 22 July 2024). Wider Person dataset: http://www.cbsr.ia.ac.cn/users/sfzhang/WiderPerson/ (accessed on 22 July 2024).
